# 3D Printing of Metal/Metal Oxide Incorporated Thermoplastic Nanocomposites With Antimicrobial Properties

**DOI:** 10.3389/fbioe.2020.568186

**Published:** 2020-09-15

**Authors:** Turdimuhammad Abdullah, Rayyan O. Qurban, Sherifdeen O. Bolarinwa, Ahmed A. Mirza, Mirza Pasovic, Adnan Memic

**Affiliations:** ^1^Center of Nanotechnology, King Abdulaziz University, Jeddah, Saudi Arabia; ^2^Department of Biochemistry, Faculty of Science, King Abdulaziz University, Jeddah, Saudi Arabia; ^3^Department of Physics, Faculty of Science, King Abdulaziz University, Jeddah, Saudi Arabia; ^4^Department of Medical Laboratory Technology, Faculty of Applied Medical Sciences, King Abdulaziz University, Jeddah, Saudi Arabia; ^5^Department of Electrical and Computer Engineering, Faculty of Engineering, King Abdulaziz University, Jeddah, Saudi Arabia

**Keywords:** 3D printing, antimicrobial, metals, metal oxides, thermoplastics, nanocomposites

## Abstract

Three-dimensional (3D) printing has experienced a steady increase in popularity for direct manufacturing, where complex geometric items can be produced without the aid of templating tools, and manufacturing waste can be remarkably reduced. While customized medical devices and daily life items can be made by 3D printing of thermoplastics, microbial contamination has been a serious obstacle during their usage. A very clever approaches to overcome this challenge is to incorporate antimicrobial metal or metal oxide (M/MO) nanoparticles within the thermoplastics during or prior to 3D printing. Many M/MO nanoparticles can prevent contamination from a wide range of microorganism, including antibiotic-resistant bacteria via various antimicrobial mechanisms. Additionally, they can be easily printed with thermoplastic without losing their integrity and functionality. In this mini review, we summarize recent advancements and discuss future trends related to the development of 3D printed antimicrobial thermoplastic nanocomposites by addition of M/MO nanoparticles.

## Introduction

Three-dimensional (3D) printing has garnered great interest among not only manufacturers for quick prototyping but also amongst the general public (MacDonald and Wicker, 2016). According to Wohlers Report 2020, the size of the global 3D printing market is $13.7 billion, exhibiting 25% annual growth since 2014 (Wohlers Talk, 2020). The technology allows for end-use products with complex shapes to be precisely fabricated by a layered additive approach without templating tools such as molds (MacDonald and Wicker, 2016; Valino et al., 2019). Accordingly, customized production using this technique can remarkably reduce the cost for low-volume manufacturing and even provide opportunities for customers to make their own “mini factory” (Gibson et al., 2014; Memic et al., 2017; Poelma and Rolland, 2017). Moreover, traditional subtractive manufacturing causes massive materials waste, which can be reduced by as much as 90% when 3D printing is utilized (Valino et al., 2019). Due to these advantages, 3D printing has become a promising alternative for producing aerospace, automotive, healthcare and consumer devices (MacDonald and Wicker, 2016; Martin et al., 2017; Gerdes et al., 2020). However, current 3D printing potential is hampered by limited material choices that meet required performance specifications for a particular application (MacDonald and Wicker, 2016). Therefore, developing multifunctional advanced materials that can easily satisfy end-use needs has become one of the central foci for researchers in this field.

Thermoplastics can be inexpensive, easy to process, chemically stable, lightweight, and flexible materials making them very attractive for 3D printing applications (Poelma and Rolland, 2017; Blok et al., 2018). Many personal and medical devices are mainly made of thermoplastics (Blok et al., 2018; Valino et al., 2019), making them the main material source for customer-level desktop printing systems. Furthermore, they are easily used in 3D printing applications, and are printable via different approaches such as selective laser sintering (SLS), stereolithography (SLA) and fused deposition modeling (FDM). Among them, FDM is one of the most popular and cost-effective printing techniques and is almost unique for printing of thermoplastics (Valino et al., 2019). However, microbial contamination still represent a challenge for biomedical applications of 3D printing, especially for handheld and medical devices (González-Henríquez et al., 2019a; Memic et al., 2019). In general, contamination associated with medical devices has been a serious issue in clinical treatment. Bacterial contamination can pose a critical threat to patients and considerably increase the healthcare cost due to need for reoperation and/or replacement of infected devices (Vasilev et al., 2009; Tamayo et al., 2016). For example, urinary catheters, indwelling vascular catheters and mechanical ventilation are responsible for 95% of urinary tract infections, 87% of bloodstream infections and 86% of pneumonia cases, respectively (Richards et al., 1999; Weinstein and Darouiche, 2001). Accumulation of bacteria on polymeric substrates could cause the formation of biofilms in which bacteria are 1,000 to 10,000 times more resistant to antibiotics than those not in a biofilm (Vasilev et al., 2009; Gnanadhas et al., 2015). Thus, it is vital to develop 3D printable antimicrobial thermoplastic materials to minimize risk of infection during their usage.

The current standard to fight infections, namely the usage of antibiotics, is facing a challenge attributed to ongoing mutation of bacterial microorganisms. As mutations are constantly occurring, so is the development of resistance to antibiotics (Gold et al., 2018). Alternatively, integration of metals and metal oxides (M/MO) within thermoplastics is one of the most promising approaches to design flexible plastic devices with antimicrobial properties (Emamifar, 2011; Tamayo et al., 2016). Most of the essential metals such as copper, zinc, magnesium and their oxides have strong biocidal effects, while other non-essential M/MO including silver, gold and cerium oxide are popular antibacterial agents (Al-Shawafi et al., 2017; Gold et al., 2018). Recent advancements in nanotechnology have enabled the synthesis of M/MO in the nano-size range, which greatly enhance their antimicrobial performance (Martinez-Gutierrez et al., 2010; Gold et al., 2018). Low concentrations, ranging from 0.1 to 4% of M/MO nanoparticles added in the polymer matrix can be functionally sufficient for prevention of most infections; a concentration often compatible with mammalian cells with negligible toxicity (Martinez-Gutierrez et al., 2010; Diez-Pascual and Diez-Vicente, 2017). Additionally M/MO nanoparticles inhibit or stunt bacterial growth via various mechanisms dissimilar to antibiotics, which would require a specific type of bacteria to undergo multiple gene mutations to generate any kind of resistance, if any at all (Gold et al., 2018). Furthermore, M/MO do not chemically deteriorate or thermally degrade in the temperature range of 3D printing of thermoplastics (Rhim et al., 2013; Palza, 2015). To best of our knowledge, this is one of the first reviews to summarize the progress made in this sub-field.

In this mini review, we will summarize the latest progress, advances and trends related to M/MO induced thermoplastic composites fabricated using 3D printing. First, we will discuss general aspects of the antimicrobial mechanism of thermoplastic nanocomposites. Next, we will describe the incorporation of nanoparticles in 3D printing and discuss key factors affecting antimicrobial activity of thermoplastic composites. Finally, we will highlight the impact of 3D printed antimicrobial thermoplastics in different applications and give insights for future developments. The overall purpose of this mini review is to highlight the impact of antimicrobial thermoplastic composites on progressive application of 3D printing.

## Antimicrobial Mechanism of the Nanocomposites

Antimicrobial activity of metals such as silver and copper has been observed since ancient times, as they were used for water sterilization and wound healing (Palza, 2015; Tamayo et al., 2016). However, the broad potential of M/MO antimicrobial agents has more recently been recognized due to the advancements in nanotechnology (Hasan et al., 2018). Metals and their oxides can form nanoparticles through numerous top-down and bottom-top synthesis approaches including sol-gel, co-precipitation, electrochemical, green synthesis, microwave, and sonochemical amongst other approaches (Salah et al., 2019). M/MO nanoparticles exhibit significantly superior antibacterial performance compared to the micro/macro particles or the bulk surface, often correlated with their smaller size and their high surface area that aid in their bioactivity and mechanism of action discussed below (Gold et al., 2018). Additionally, M/MO nanoparticles have advantages including the prevention of biofilm formation by inhibiting planktonic growth, altering lipids and proteins, damaging DNA, and interfering with enzyme activities of bacteria (Gold et al., 2018). Hence, antibiotic-resistant bacteria could be effectively inhibited by M/MO nanoparticles, as they exert antimicrobial activities via other mechanisms different from ones exerted by antibiotics (Raghunath and Perumal, 2017). However, over-dosage and/or an immediate release of the nanoparticles could cause injuries to certain eukaryotic cells (Gold et al., 2018).

Incorporation of M/MO nanoparticles into polymers could improve overall antibacterial efficacy of the nanocomposite, due to the synergetic polymer and nanoparticle effects (Tamayo et al., 2016). In most M/MO thermoplastic nanocomposites, the antimicrobial mechanism is mainly associated with toxicity of M/MO nanoparticles (Sánchez-López et al., 2020). On the other hand, polymer matrix as a support regulates the release behavior of the nanoparticles and ions as a response to bacterial adhesion on the composite surface (Supplementary Figure S1). Namely, water molecules could diffuse from the bacterial medium into the polymer matrix, and cause release of metal ions. Although antimicrobial mechanisms of M/MO are still a subject of meticulous research, owing to the continuous mutation of bacteria, there are three major mechanisms (Gold et al., 2018).

### Metal Ion Limitation

Metal and metal oxide nanoparticles can inhibit bacterial growth by attacking the electronegative phospholipid bilayers of the bacterial cell wall with electropositive metal ions (Chandrangsu et al., 2017; Raghunath and Perumal, 2017). They release cations to the anionic sites of the cell membrane and neutralize charges within the cell via electrostatic interaction. This charge difference favors accumulation of metallic ions, which in turn permeates the cell membrane and recruit intrinsic metals to be adsorbed into it (Raghunath and Perumal, 2017). In response, the homeostatic system in the cell releases more ions from its storage to make up for the loss. This cycle continues until the cell is starved of its essential metals and metal-dependent metabolic processes come to a halt (Gold et al., 2018). This antimicrobial mechanism has been demonstrated in iron oxide and zinc-based nanoparticles (Raghunath and Perumal, 2017).

### Biomolecular Interaction

Another bactericidal mechanism of M/MO nanoparticles is through interaction with DNA and cytosolic enzymes of various microorganism (Raghunath and Perumal, 2017). Metals and their ions could crosslink DNA and disrupt its helical structure halting bacterial proliferation. Furthermore, metals and their ions could directly bind to enzymes and disrupt their tertiary structures, which in turn disrupt digestion and respiration processes (Imlay, 2014). As a result, bacterial cells start to lose metabolic activity and die. Metal ions like copper (I), zinc (II), manganese (II), and iron (II) have been reported to exhibit this antibacterial mechanism (Skaar and Raffatellu, 2015).

### Reactive Oxygen Species (ROS) Formation

Many transitional metals and their oxides have strong redox properties allowing them to gain electrons from reactive donor sites (Raghunath and Perumal, 2017). Hence the intermolecular interaction between M/MO nanoparticles and the bacterial cells initiates the formation of many ROS such as hydroxyl radicals (OH^•^), superoxide anion (O^–^_2_) and hydrogen peroxide (H_2_O_2_). Further propogation of ROS via different reactions increase their concentrations, causing oxidative stress (Nel et al., 2006). This stress mediates the damage of cell macromolecules, distortion of proteins and lipids, alteration of DNA/RNA, inhibition of enzymes activities, and eventually death of the microbe (Gold et al., 2018). For example, silver, gold (Zheng et al., 2017), zinc oxide (He et al., 2011) and magnesium oxide have been reported to prompt ROS formation (Gold et al., 2018).

## 3D Printing of Thermoplastic Nanocomposites

In general, 3D printing is a process to build a structure by adding materials layer-by-layer according to a computer aided design (CAD) model (Jiménez et al., 2019). Initially a CAD model can be created by various 3D design software and stored in a format adapted for 3D printing (Valino et al., 2019). Afterwards, the designed model is transferred to slicer programs to set the printing options such as the thickness of the model, the height of the layers, filling ratio and printing speed, which can be saved as a G-code file (Jiménez et al., 2019). Finally, various types of 3D printers use the G-code file to build the models to be ready for post-manufacturing removal of supports, sanding and filing processes (Guo et al., 2019).

**FIGURE 1:**
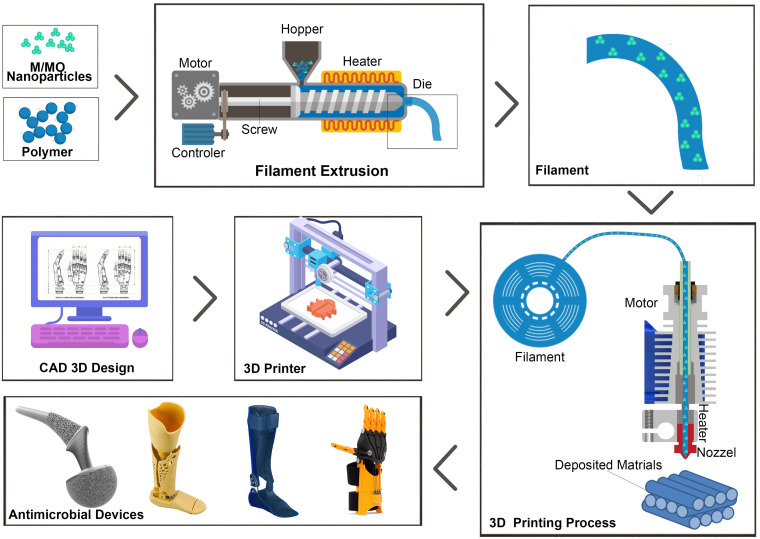
Schematic illustration of 3D printing of M/MO incorporated thermoplastic nanocomposites using FDM process. Composite filaments can be made by extruding mixture of the M/MO nanoparticles with the thermoplastics. Then filaments are heat-melted and extruded from a nozzle and allowed to solidify in a printing bed. Pre-programmed computer aided design (CAD) model is used to control the printing process.

Fused deposition modeling (FDM), one of the most common 3D printing techniques, applies thermoplastic based polymers and nanocomposites (Figure 1). In this technique, filaments are heat-melted and extruded from a nozzle and allowed to solidify in a printing bed. The hot nozzle commonly moves in X and Y direction to build the first layer, while succeeding layers are continuously being built by moving the nozzle up in the Z direction (Ngo et al., 2018; Jiménez et al., 2019). Addition of M/MO nanoparticles is usually achieved during formation of filaments prior to 3D printing. Melt-blending, solvent-casting and surface coating (Supplementary Figure S2) are few of the approaches to mix the nanoparticles with thermoplastics (González-Henríquez et al., 2019b).

Another approach to print thermoplastic nanocomposites is SLS, in which a laser selectively sinters the particles of a polymer powder and fuses them together, building the intended part layer-by-layer (Mazzoli, 2013). The process starts with the powder and the build area being heated to just near the melting temperature of the polymer. Then, a recoating blade spreads a thin layer of powder over the build platform (Paul and Anand, 2012). Next, a laser fuses powder polymers together. After scanning each cross-section of the component, the power bed is lowered down to the next layer, and another layer is built on top. This process is repeated until the intended model is completed (Paul and Anand, 2012; Valino et al., 2019). For this approach, addition of powder M/MO nanoparticles with the polymer powder is done by mechanical mixers such as rotary tumblers (Turner et al., 2020). The mixing process is easier and can result in a more uniform distribution of nanoparticles when compared to FDM. Yet another method utilized to create antibacterial thermoplastic nanocomposite 3D printed models is SLA. In this method, M/MO nanoparticles are first dispersed into the monomer solution prior to its polymerization (Totu et al., 2017). The resin is then deposited layer-by-layer based on the designed model. In final step the polymerization is effectuated by UV or other light sources (Weng et al., 2016). In addition, liquid deposition molding (LDM), solvent-cast 3D printing (SCP) and melt electrowriting (MEW) can also be used to print the thermoplastic nanocomposites (Hewitt et al., 2019; Nonato et al., 2019). However, regardless of the printing approach and method of nanocomposite preparation much research remains to be done.

## Factors Influencing Antimicrobial Properties of Nanocomposites

As discussed above, M/MO nanoparticles can play a key role in the antibacterial properties of the thermoplastic nanocomposites, in which, their size, ionic status and concentration, strongly influence the antimicrobial performance of the nanocomposite (Salah et al., 2020). Moreover, ionic release of M/MO nanoparticles is critical to the balance between immediate antimicrobial activity and long-term efficacy of the nanocomposites. Accordingly, polymer properties affecting the ionic release of M/MO nanoparticles such as hydrophilicity, density and crystallinity should be carefully considered in the design of antimicrobial nanocomposites for different applications (Tamayo et al., 2016).

Antimicrobial performance of nanocomposites can significantly differ depending on the M/MO incorporated into the same polymer matrix. For example, Muwaffak et al. (2017) suggests that silver in polycaprolactone (PCL) exhibits a superior antibacterial effect against *Staphylococcus aureus* when compared to copper or zinc. Studies also showed that antibacterial activity of the nanocomposite can be greatly enhanced by size reduction of these nanoparticles. For example, polylactic acid (PLA) filled with 10% silver wires can inhibit only 50% of *Escherichia coli* growth when the wire diameter is 330 nm (Walker et al., 2020), while 100% inhibition of the same bacterial strain can be achieved by adding only 1% silver nanowires with the average diameter of 60 nm (Bayraktar et al., 2019). Furthermore, oxidized or ionized metal particles are recommended for higher antibacterial efficacy over pure metal particles. For instance, Delgado et al. (2011) investigated the effect of adding copper or copper oxide on antimicrobial activity of polypropylene nanocomposite. Their study revealed that copper oxide fillers are much more efficient at eliminating bacteria when compared to copper fillers, when tested against *Escherichia coli* (Delgado et al., 2011). Finally, increasing concentration of the antibacterial nanoparticles is a common strategy to enhance overall antibacterial efficiency of the nanocomposites (Kalakonda et al., 2017). Nonetheless, it is important to consider other effects of the printed nanocomposites, such as biocompatibility and environmental safety according to their potential applications.

## Applications

3D printed antimicrobial thermoplastic nanocomposites have ample application potential in the medical and healthcare fields, as they can provide an on-demand support for the customization and personalization of medical devices (Zuniga and Cortes, 2020). Specifically, they are very attractive for surgical prosthesis since some of the thermoplastics (e.g., PLA, PCL) have been extensively studied for tissue engineering and regenerative medicine application (Table 1). With the integration of M/MO nanoparticles, bacterial infection can be avoided during surgical implantation, which otherwise could affect tissue growth, postpone surgical recovery, upsurge risks of complications, and even cause death (Abudula et al., 2020). For example, Zou et al. (2020) prepared nanocomposite scaffolds containing poly(lactic-co-glycolic acid) (PLGA), copper/zinc based zeolitic-imidazolate-frameworks (Cu@ZIP-8) by FDM-based 3D printing for infected bone repair application. They suggested that a sustainable release of Cu@ZIP-8 in aqueous media ensures long-term antibacterial efficacy of the developed scaffolds (Zou et al., 2020). Afghah et al. (2020) developed a PCL-poly propylene succinate (PCL-PPSu) composite with silver-doped biocidal properties, which can be printable by FDM. They suggested that the printed scaffolds can be potentially applied for skin tissue engineering due to their inhibition effects against various microorganism, a good cyto-compatibility and biodegradability (Afghah et al., 2020). Similarly, Totu et al. (2017) developed an antibacterial poly methylmethacrylate/TiO_2_ nanocomposite that was employed for the fabrication of complete denture sets prototyped by SLA 3D printing. Although the composite only contained 0.4% of TiO_2_ nanoparticles, it exhibited sufficient antimicrobial activity against *Candida* species. They also tested these nanocomposites with real patients and demonstrated that this manufacturing approach is a promising treatment option for patients diagnosed with complete edentulism (Totu et al., 2017; Cristache et al., 2020). Turner et al. (2020) introduced a SLS printing of antibacterial nanocomposite by mixing polyamide 12 with 1% of commercially available B65003 silver additives to make delicate, complex, and personalized devices for medical and healthcare applications (Turner et al., 2020).

**TABLE 1:** Examples of 3D printing of metal/metal oxide incorporated thermoplastic nanocomposites with antibacterial properties for various applications.

Materials			Printing method	Application	References
Thermoplastic	M/MO	Other(s)			
PLGA	Copper	ZIF-8	HT-LDM	Infected bone repair	Zou et al. (2020)
PMMA	Titanium oxide	–	SLA	Digital dentistry	Cristache et al. (2020)
PLLA	Silver	HNTs	SLS	Infected bone repair	Guo et al. (2020)
PCL-PPSu	Silver	–	FDM	Skin tissue engineering	Afghah et al. (2020)
ABS	Zinc	–	FDM + casting	Infected bone repair	Cockerill et al. (2020)
Polyamide 12	Silver	–	SLS	Personalized devices	Turner et al. (2020)
PLA	Copper-zinc alloy	PWF	FDM	Handled devices	Yang et al. (2020)
PLA/PGA	Silver	MBG	SLS	Infected bone repair	Shuai et al. (2019)
PLA	Zinc oxide		SCP	Food packaging	Nonato et al. (2019)
PCL	Copper	Bio glass	LDM	Infected bone repair	Wang et al. (2019)
PLA	Copper		FDM	Finger prosthesis	Young et al. (2019)
PLA	Silver	–	FDM	Public health	Bayraktar et al. (2019)
PLA/PGA	Silver	Grapheme	SLS	Bone tissue engineering	Shuai et al. (2018)
PLA	Copper		FDM	Finger prosthesis	Zuniga (2018)
PMMA	Titanium oxide	–	SLA	Digital dentistry	Totu et al. (2017)
PBAT/PLA	Silver	Egg-shell	FDM	Food packaging	Tiimob et al. (2017)
ABS	Zinc oxide	–	FDM	Toys	León-Cabezas et al. (2017)

*PLGA, Poly(lactic-co-glycolic acid); PMMA, Poly(methyl methacrylate); PLLA, Poly-L-lactide; PCL, Poly (caprolactone); PPSu, Poly propylene succinate; ABS, Acrylonitrile butadiene styrene; PLA, Poly (lactic acid); PGA, Polyglycolide; PBAT, Poly(butylene adipate-co-terephthalate); M/MO, Metal/metal oxide; ZIF-8, Zinc based zeolitic-imidazolate-frameworks; HNTs, Halloysite nanotubes; PWF, Particleboard wood flour; MBG, Mesoporous bioactive glass; HT, High temperature; LDM, Liquid deposition modeling; SLA, Stereolithography; SLS, Selective laser sintering; FDM, Fused deposition modeling; SCP, Solvent-cast 3D printing.*

Likewise, tuning the mechanical properties by incorporation of M/MO nanoparticles could be of great benefit especially to the food-packaging industry. More specifically, nanocomposites can be a versatile alternative to traditionally manufactured plastics for reducing food contamination and provide safer degradable options for packaging. For example, Tiimob et al. (2017) incorporated silver nanoparticles into a polymer blend composed of 70% poly butylene-co-adipate-terephthalate and 30% PLA to design 3D printed nanocomposites by FDM. Their research suggested that the developed composites can be used for food packaging due to their robust mechanical properties and ability to control growth of microorganisms in food (Tiimob et al., 2017).

Finally, 3D printed antibacterial nanocomposites can also be widely integrated in daily-life products (Huang et al., 2016). Many of the devices and tools that are used routinely in day-to-day tasks can be made with antibacterial materials to reduce the potential negative effects of pathogenic bacteria and fungi. Notably, objects associated with public use can be made safer by 3D printing with antimicrobial nanocomposites. Yang et al. (2020) fabricated a particleboard wood flour/PLA composite filament reinforced by copper-zinc alloy nanoparticles for FDM-based 3D printing. They suggested that the developed filament can be used to print personalized furniture, public benches and toys, owing to its good antimicrobial properties and environmental safety (Yang et al., 2020). In short, these examples provide a clear vision of how 3D printing of antimicrobial nanocomposites can be applied to make imaginative and customized products.

## Challenges and Future Scope

In conclusion, M/MO nanoparticles as antimicrobial fillers can greatly increase the value of 3D printed thermoplastic products. Nonetheless, challenges remain in commercializing these thermoplastic nanocomposites for a wider range of applications. For example, one of the critical issues related to their clinical application is that many antibacterial tests have been performed *in vitro*, in which some factors that influence antibacterial performance *in vivo*, such as inflammation, are neglected (Wang et al., 2016; Jiao et al., 2019). A similar challenge is having comprehensive compatibility testing of these nanocomposites that are essential for their use with the human body. Long-term exposure and biosafety remain a major challenge when consider their application in the medical field, particularly toxic effect of M/MO on a wide range of cells and tissue need to be assessed. For example, Ickrath et al. (2017) suggested cytotoxic and genotoxic effects of zinc oxide nanoparticles after long-term and repetitive exposure to human mesenchymal stem cells. Therefore, currently reported short-term, single cell-line reference data is often not enough to provide a generalized biocompatibility profile of the developed materials in many if not most cases (Tamayo et al., 2016; Rtimi et al., 2019). In addition, burst-release of antimicrobial agents (i.e., metal ions or particles) can represent a significant limitation of the 3D printed composites. One way to overcome this challenge would be by entrapping antibacterial particles with an additional polymer layer using coaxial nozzle configurations (Yao et al., 2019). Meanwhile, release properties of antibacterial components could be also controlled by stimuli-responsive materials, especially if the nanocomposites can be release in response to bacterial adhesion to the material (González-Henríquez et al., 2019b). In addition, controlled release of nanoparticles as ions or metallic particles from the polymer matrix is often key to maintaining the balance between antimicrobial performance and mammalian cytocompatibility (Tamayol et al., 2017; Mas-Moruno et al., 2019).

Other challenges include addition of M/MO nanoparticles into 3D printed thermoplastics often through a multistage process. For example, the nanoparticles first need to be synthesized, then homogeneously dispersed into the polymer matrix and finally 3D printed. This not only increases the total manufacturing cost, but can also deteriorate 3D print quality by inducing filler defects, poor adhesion and particle agglomeration or uneven distribution. Alternatively, *in situ* synthesis of nanoparticles could possibly be a way to overcome such challenges (Palza, 2015). The concept of *in situ* printing has recently emerged, in which personalized healthcare devices and prosthesis can be directly printed on the defected tissue or organ without 3D scanning and computational design. Particularly, robotic assisted *in situ* printing could be one of the most exciting techniques introduced in the future, which would elevate novelty of 3D printing to a new level (Ma et al., 2020). Finally, there are many possible application areas yet to be studied using 3D printed nanocomposites, particularly considering their capacity to tailor the product design on a personal level (Ishack and Lipner, 2020). Specifically, there is a lot of potential for developing personal protective equipment, including masks, ventilators tubes, gloves, which could be especially important during the current global pandemic (Zuniga and Cortes, 2020).

## Author Contributions

The manuscript was written and edited with contribution from all authors. All authors have given approval to the final version of the manuscript.

## Conflict of Interest

The authors declare that the research was conducted in the absence of any commercial or financial relationships that could be construed as a potential conflict of interest.

## References

[B1] Abudula T. Gauthaman K. Hammad A. H. Joshi Navare K. Alshahrie A. A. Bencherif S. A. (2020). Oxygen-releasing antibacterial nanofibrous scaffolds for tissue engineering applications. *Polymers* 12:1233. 10.3390/polym12061233 32485817 PMC7361702

[B2] Afghah F. Ullah M. Zanjani J. S. M. Sut P. A. Sen O. Emanet M. (2020). 3D printing of silver-doped polycaprolactone-poly (propylene succinate) composite scaffolds for skin tissue engineering. *Biomed. Mater.* 15:035015. 10.1088/1748-605X/ab7417 32032966

[B3] Al-Shawafi W. M. Salah N. Alshahrie A. Ahmed Y. M. Moselhy S. S. Hammad A. H. (2017). Size controlled ultrafine CeO2 nanoparticles produced by the microwave assisted route and their antimicrobial activity. *J. Mater. Sci.* 28:177. 10.1007/s10856-017-5990-5998 28956214

[B4] Bayraktar I. Doganay D. Coskun S. Kaynak C. Akca G. Unalan H. E. (2019). 3D printed antibacterial silver nanowire/polylactide nanocomposites. *Composit. Part B Eng.* 172 671–678. 10.1016/j.compositesb.2019.05.059

[B5] Blok L. G. Longana M. L. Yu H. Woods B. K. (2018). An investigation into 3D printing of fibre reinforced thermoplastic composites. *Add. Manufact.* 22 176–186. 10.1016/j.addma.2018.04.039

[B6] Chandrangsu P. Rensing C. Helmann J. D. (2017). Metal homeostasis and resistance in bacteria. *Nat. Rev. Microbiol.* 15:338. 10.1038/nrmicro.2017.15 28344348 PMC5963929

[B7] Cockerill I. Su Y. Sinha S. Qin Y.-X. Zheng Y. Young M. L. (2020). Porous zinc scaffolds for bone tissue engineering applications: a novel additive manufacturing and casting approach. *Mater. Sci. Eng. C* 110:110738. 10.1016/j.msec.2020.110738 32204047 PMC7096330

[B8] Cristache C. M. Totu E. E. Iorgulescu G. Pantazi A. Dorobantu D. Nechifor A. C. (2020). Eighteen months follow-up with patient-centered outcomes assessment of complete dentures manufactured using a hybrid nanocomposite and additive CAD/CAM protocol. *J. Clin. Med.* 9:324. 10.3390/jcm9020324 31979345 PMC7073708

[B9] Delgado K. Quijada R. Palma R. Palza H. (2011). Polypropylene with embedded copper metal or copper oxide nanoparticles as a novel plastic antimicrobial agent. *Lett. Appl. Microbiol.* 53 50–54. 10.1111/j.1472-765x.2011.03069.x 21535046

[B10] Diez-Pascual A. M. Diez-Vicente A. L. (2017). Antibacterial SnO2 nanorods as efficient fillers of poly (propylene fumarate-co-ethylene glycol) biomaterials. *Mater. Sci. Eng. C* 78 806–816. 10.1016/j.msec.2017.04.114 28576053

[B11] Emamifar A. (2011). “Applications of antimicrobial polymer nanocomposites in food packaging,” *Advances in Nanocomposite Technology*, 299–318.

[B12] Gerdes S. Mostafavi A. Ramesh S. Memic A. Rivero I. V. Rao P. (2020). Process-structure-quality relationships of three-dimensional printed poly (Caprolactone)-hydroxyapatite scaffolds. *Tissue Eng. Part A* 26 279–291. 10.1089/ten.tea.2019.0237 31964254 PMC7366318

[B13] Gibson I. Rosen D. W. Stucker B. (2014). *Additive Manufacturing Technologies.* Berlin: Springer.

[B14] Gnanadhas D. P. Elango M. Janardhanraj S. Srinandan C. Datey A. Strugnell R. A. (2015). Successful treatment of biofilm infections using shock waves combined with antibiotic therapy. *Sci. Rep.* 5:17440. 10.1038/srep17440 PMC4674795 26658706

[B15] Gold K. Slay B. Knackstedt M. Gaharwar A. K. (2018). Antimicrobial activity of metal and metal-oxide based nanoparticles. *Adv. Therap.* 1:1700033. 10.1002/adtp.201700033

[B16] González-Henríquez C. M. Sarabia-Vallejos M. A. Rodríguez Hernandez J. (2019a). Antimicrobial polymers for additive manufacturing. *Intern. J. Mol. Sci.* 20:1210. 10.3390/ijms20051210 30857355 PMC6429148

[B17] González-Henríquez C. M. Sarabia-Vallejos M. A. Rodriguez-Hernandez J. (2019b). Polymers for additive manufacturing and 4D-printing: materials, methodologies, and biomedical applications. *Prog. Poly. Sci.* 94 57–116. 10.1016/j.progpolymsci.2019.03.001

[B18] Guo H. Lv R. Bai S. (2019). Recent advances on 3D printing graphene-based composites. *Nano Mater. Sci.* 1 101–115. 10.1016/j.nanoms.2019.03.003

[B19] Guo W. Liu W. Xu L. Feng P. Zhang Y. Yang W. (2020). Halloysite nanotubes loaded with nano silver for the sustained-release of antibacterial polymer nanocomposite scaffolds. *J. Mater. Sci. Technol.* 46 237–247. 10.1016/j.jmst.2019.11.019

[B20] Hasan A. Morshed M. Memic A. Hassan S. Webster T. J. Marei H. E.-S. (2018). Nanoparticles in tissue engineering: applications, challenges and prospects. *Intern. J. Nanomed.* 13:5637. 10.2147/ijn.s153758 30288038 PMC6161712

[B21] He L. Liu Y. Mustapha A. Lin M. (2011). Antifungal activity of zinc oxide nanoparticles against *Botrytis cinerea* and *Penicillium expansum*. *Microbiol. Res.* 166 207–215. 10.1016/j.micres.2010.03.003 20630731

[B22] Hewitt E. Mros S. McConnell M. Cabral J. D. Ali A. (2019). Melt-electrowriting with novel milk protein/PCL biomaterials for skin regeneration. *Biomed. Mater.* 14:055013. 10.1088/1748-605X/ab3344 31318339

[B23] Huang K.-S. Yang C.-H. Huang S.-L. Chen C.-Y. Lu Y.-Y. Lin Y.-S. (2016). Recent advances in antimicrobial polymers: a mini-review. *Intern. J. Mol. Sci.* 17:1578. 10.3390/ijms17091578 27657043 PMC5037843

[B24] Ickrath P. Wagner M. Scherzad A. Gehrke T. Burghartz M. Hagen R. (2017). Time-dependent toxic and genotoxic effects of zinc oxide nanoparticles after long-term and repetitive exposure to human mesenchymal stem cells. *Intern. J. Environ. Res. Public Health* 14:1590. 10.3390/ijerph14121590 29258234 PMC5751007

[B25] Imlay J. A. (2014). The mismetallation of enzymes during oxidative stress. *J. Biol. Chem.* 289 28121–28128. 10.1074/jbc.r114.588814 25160623 PMC4192467

[B26] Ishack S. Lipner S. R. (2020). Applications of 3D printing technology to address COVID-19 related supply shortages. *Am. J. Med.* 133 771–773. 10.1016/j.amjmed.2020.04.002 32330492 PMC7172844

[B27] Jiao Y. Tay F. R. Niu L.-N. Chen J.-H. (2019). Advancing antimicrobial strategies for managing oral biofilm infections. *Intern. J. Oral Sci.* 11 1–11. 10.1038/ijos.2014.65 31570700 PMC6802668

[B28] Jiménez M. Romero L. Domínguez I. A. Espinosa M. D. M. Domínguez M. (2019). Additive manufacturing technologies: an overview about 3D printing methods and future prospects. *Complexity* 2019 1–30. 10.1155/2019/9656938

[B29] Kalakonda P. Aldhahri M. A. Abdel-Wahab M. S. Tamayol A. Moghaddam K. M. Rached F. B. (2017). Microfibrous silver-coated polymeric scaffolds with tunable mechanical properties. *RSC Adv.* 7 34331–34338. 10.1039/c6ra25151j PMC5388165 28336896

[B30] León-Cabezas M. A. Martínez-García A. Varela-Gandía F. J. (2017). Innovative functionalized monofilaments for 3D printing using fused deposition modeling for the toy industry. *Proc. Manufact.* 13 738–745. 10.1016/j.promfg.2017.09.130

[B31] Ma K. Zhao T. Yang L. Wang P. Jin J. Teng H. (2020). Application of robotic-assisted in situ 3D printing in cartilage regeneration with HAMA hydrogel: an in vivo study. *J. Adv. Res.* 23 123–132. 10.1016/j.jare.2020.01.010 32099674 PMC7030996

[B32] MacDonald E. Wicker R. (2016). Multiprocess 3D printing for increasing component functionality. *Science* 353:aaf2093. 10.1126/science.aaf2093 27708075

[B33] Martin J. H. Yahata B. D. Hundley J. M. Mayer J. A. Schaedler T. A. Pollock T. M. (2017). 3D printing of high-strength aluminium alloys. *Nature* 549:365. 10.1038/nature23894 28933439

[B34] Martinez-Gutierrez F. Olive P. L. Banuelos A. Orrantia E. Nino N. Sanchez E. M. (2010). Synthesis, characterization, and evaluation of antimicrobial and cytotoxic effect of silver and titanium nanoparticles. *Nanomed. Nanotechnol. Biol. Med.* 6 681–688. 10.1016/j.nano.2010.02.001 20215045

[B35] Mas-Moruno C. Su B. Dalby M. J. (2019). Multifunctional coatings and nanotopographies: toward cell instructive and antibacterial implants. *Adv. Healthc. Mater.* 8:1801103. 10.1002/adhm.201801103 30468010

[B36] Mazzoli A. (2013). Selective laser sintering in biomedical engineering. *Med. Biol. Eng. Comput.* 51 245–256. 10.1007/s11517-012-1001-x 23250790

[B37] Memic A. Abudula T. Mohammed H. S. Joshi Navare K. Colombani T. Bencherif S. A. (2019). Latest progress in electrospun nanofibers for wound healing applications. *ACS Appl. Bio. Mater.* 2 952–969. 10.1021/acsabm.8b00637 35021385

[B38] Memic A. Navaei A. Mirani B. Cordova J. A. V. Aldhahri M. Dolatshahi-Pirouz A. (2017). Bioprinting technologies for disease modeling. *Biotechnol. Lett.* 39 1279–1290. 10.1007/s10529-017-2360-z 28550360

[B39] Muwaffak Z. Goyanes A. Clark V. Basit A. W. Hilton S. T. Gaisford S. (2017). Patient-specific 3D scanned and 3D printed antimicrobial polycaprolactone wound dressings. *Int. J. Pharm.* 527 161–170. 10.1016/j.ijpharm.2017.04.077 28461267

[B40] Nel A. Xia T. Mädler L. Li N. (2006). Toxic potential of materials at the nanolevel. *Science* 311 622–627. 10.1126/science.1114397 16456071

[B41] Ngo T. D. Kashani A. Imbalzano G. Nguyen K. T. Q. Hui D. (2018). Additive manufacturing (3D printing): a review of materials, methods, applications and challenges. *Composit. Part B Eng.* 143 172–196. 10.1016/j.compositesb.2018.02.012

[B42] Nonato R. C. Mei L. H. I. Bonse B. C. Chinaglia E. F. Morales A. R. (2019). Nanocomposites of PLA containing ZnO nanofibers made by solvent cast 3D printing: production and characterization. *Eur. Poly. J.* 114 271–278. 10.1016/j.eurpolymj.2019.02.026

[B43] Palza H. (2015). Antimicrobial polymers with metal nanoparticles. *Intern. J. Mol. Sci.* 16 2099–2116. 10.3390/ijms16012099 25607734 PMC4307351

[B44] Paul R. Anand S. (2012). Process energy analysis and optimization in selective laser sintering. *J. Manufact. Syst.* 31 429–437. 10.1016/j.jmsy.2012.07.004

[B45] Poelma J. Rolland J. (2017). Rethinking digital manufacturing with polymers. *Science* 358 1384–1385. 10.1126/science.aaq1351 29242332

[B46] Raghunath A. Perumal E. (2017). Metal oxide nanoparticles as antimicrobial agents: a promise for the future. *Int. J. Antimicrob. Agents* 49 137–152. 10.1016/j.ijantimicag.2016.11.011 28089172

[B47] Rhim J.-W. Park H.-M. Ha C.-S. (2013). Bio-nanocomposites for food packaging applications. *Prog. Poly. Sci.* 38 1629–1652.

[B48] Richards M. J. Edwards J. R. Culver D. H. Gaynes R. P. (1999). Nosocomial infections in medical intensive care units in the United States. *Crit. Care Med.* 27 887–892. 10.1097/00003246-199905000-00020 10362409

[B49] Rtimi S. Dionysiou D. D. Pillai S. C. Kiwi J. (2019). Advances in catalytic/photocatalytic bacterial inactivation by nano Ag and Cu coated surfaces and medical devices. *Appl. Catal. B Environ.* 240 291–318. 10.1016/j.apcatb.2018.07.025

[B50] Salah N. Alfawzan A. M. Allafi W. Baghdadi N. Saeed A. Alshahrie A. (2020). Size-controlled, single-crystal CuO nanosheets and the resulting polyethylene-carbon nanotube nanocomposite as antimicrobial materials. *Polym. Bull.* 1–21.

[B51] Salah N. Al-Shawafi W. M. Alshahrie A. Baghdadi N. Soliman Y. M. Memic A. (2019). Size controlled, antimicrobial ZnO nanostructures produced by the microwave assisted route. *Mater. Sci. Eng. C* 99 1164–1173. 10.1016/j.msec.2019.02.077 30889650

[B52] Sánchez-López E. Gomes D. Esteruelas G. Bonilla L. Lopez-Machado A. L. Galindo R. (2020). Metal-based nanoparticles as antimicrobial agents: an overview. *Nanomaterials* 10:292. 10.3390/nano10020292 PMC7075170 32050443

[B53] Shuai C. Guo W. Wu P. Yang W. Hu S. Xia Y. (2018). A graphene oxide-Ag co-dispersing nanosystem: dual synergistic effects on antibacterial activities and mechanical properties of polymer scaffolds. *Chem. Eng. J.* 347 322–333. 10.1016/j.cej.2018.04.092

[B54] Shuai C. Xu Y. Feng P. Wang G. Xiong S. Peng S. (2019). Antibacterial polymer scaffold based on mesoporous bioactive glass loaded with in situ grown silver. *Chem. Eng. J.* 374 304–315. 10.1016/j.cej.2019.03.273

[B55] Skaar E. P. Raffatellu M. (2015). Metals in infectious diseases and nutritional immunity. *Metallomics* 7 926–928. 10.1039/c5mt90021b 26017093

[B56] Tamayo L. Azócar M. Kogan M. Riveros A. Páez M. (2016). Copper-polymer nanocomposites: an excellent and cost-effective biocide for use on antibacterial surfaces. *Mater. Sci. Eng. C* 69 1391–1409. 10.1016/j.msec.2016.08.041 27612841

[B57] Tamayol A. Najafabadi A. H. Mostafalu P. Yetisen A. K. Commotto M. Aldhahri M. (2017). Biodegradable elastic nanofibrous platforms with integrated flexible heaters for on-demand drug delivery. *Sci. Rep.* 7 1–10. 28835675 10.1038/s41598-017-04749-8 PMC5569034

[B58] Tiimob B. J. Mwinyelle G. Abdela W. Samuel T. Jeelani S. Rangari V. K. (2017). Nanoengineered eggshell-silver tailored copolyester polymer blend film with antimicrobial properties. *J. Agric. Food Chem.* 65 1967–1976. 10.1021/acs.jafc.7b00133 28206760

[B59] Totu E. E. Nechifor A. C. Nechifor G. Aboul-Enein H. Y. Cristache C. M. (2017). Poly (methyl methacrylate) with TiO_2_ nanoparticles inclusion for stereolitographic complete denture manufacturing- the fututre in dental care for elderly edentulous patients? *J. Dent.* 59 68–77. 10.1016/j.jdent.2017.02.012 28223199

[B60] Turner R. D. Wingham J. R. Paterson T. E. Shepherd J. Majewski C. (2020). Use of silver-based additives for the development of antibacterial functionality in Laser Sintered polyamide 12 parts. *Sci. Rep.* 10 1–11. 31964969 10.1038/s41598-020-57686-4 PMC6972821

[B61] Valino A. D. Dizon J. R. C. Espera A. H. Chen Q. Messman J. Advincula R. C. (2019). Advances in 3D printing of thermoplastic polymer composites and nanocomposites. *Prog. Polym. Sci.* 98:101162. 10.1016/j.progpolymsci.2019.101162

[B62] Vasilev K. Cook J. Griesser H. J. (2009). Antibacterial surfaces for biomedical devices. *Expert Rev. Med. Dev.* 6 553–567. 10.1586/erd.09.36 19751126

[B63] Walker J. S. Arnold J. Shrestha C. Smith D. (2020). Antibacterial silver submicron wire-polylactic acid composites for fused filament fabrication. *Rapid Prototyp. J.* 26 32–38. 10.1108/rpj-04-2019-0100

[B64] Wang B. Han Y. Lin Q. Liu H. Shen C. Nan K. (2016). In vitro and in vivo evaluation of xanthan gum-succinic anhydride hydrogels for the ionic strength-sensitive release of antibacterial agents. *J. Mater. Chem. B* 4 1853–1861. 10.1039/c5tb02046h 32263062

[B65] Wang X. Molino B. Z. Pitkänen S. Ojansivu M. Xu C. Hannula M. (2019). 3D scaffolds of polycaprolactone/copper-doped bioactive glass: architecture engineering with additive manufacturing and cellular assessments in a coculture of bone marrow stem cells and endothelial cells. *ACS Biomater. Sci. Eng.* 5 4496–4510. 10.1021/acsbiomaterials.9b00105 33438415

[B66] Weinstein R. A. Darouiche R. O. (2001). Device-associated infections: a macroproblem that starts with microadherence. *Clin. Infect. Dis.* 33 1567–1572. 10.1086/323130 11577378

[B67] Weng Z. Zhou Y. Lin W. Senthil T. Wu L. (2016). Structure-property relationship of nano enhanced stereolithography resin for desktop SLA 3D printer. *Composit. Part A Appl. Sci. Manufact.* 88 234–242. 10.1016/j.compositesa.2016.05.035

[B68] Wohlers Talk (2020). *3D Printing And Additive Manufacturing State Of The Industry. Annual Worldwide Progress Report.* Fort Collins, CO: Wohlers Associates.

[B69] Yang F. Zeng J. Long H. Xiao J. Luo Y. Gu J. (2020). Micrometer copper-zinc alloy particles-reinforced wood plastic composites with high gloss and antibacterial properties for 3D printing. *Polymers* 12:621. 10.3390/polym12030621 32182784 PMC7182845

[B70] Yao Z.-C. Wang J.-C. Ahmad Z. Li J.-S. Chang M.-W. (2019). Fabrication of patterned three-dimensional micron scaled core-sheath architectures for drug patches. *Mater. Sci. Eng. C* 97 776–783. 10.1016/j.msec.2018.12.110 30678967

[B71] Young K. J. Pierce J. E. Zuniga J. M. (2019). Assessment of body-powered 3D printed partial finger prostheses: a case study. *3D Print. Med.* 5 1–8. 31049828 10.1186/s41205-019-0044-0 PMC6743133

[B72] Zheng K. Setyawati M. I. Leong D. T. Xie J. (2017). Antimicrobial gold nanoclusters. *ACS Nano* 11 6904–6910. 10.1021/acsnano.7b02035 28595000

[B73] Zou F. Jiang J. Lv F. Xia X. Ma X. (2020). Preparation of antibacterial and osteoconductive 3D-printed PLGA/Cu (I)@ ZIF-8 nanocomposite scaffolds for infected bone repair. *J. Nanobiotechnol.* 18 1–14. 10.1186/s12951-020-00594-6 PMC7045416 32103765

[B74] Zuniga J. M. (2018). 3D printed antibacterial prostheses. *Appl. Sci.* 8:1651. 10.3390/app8091651

[B75] Zuniga J. M. Cortes A. (2020). The role of additive manufacturing and antimicrobial polymers in the COVID-19 pandemic. *Expert Rev. Med. Dev.* 17 477–481. 10.1080/17434440.2020.1756771 32312129 PMC7196922

