# Supplementary material for: 3D Printing of Metal/Metal Oxide Incorporated Thermoplastic Nanocomposites With Antimicrobial Properties
Source: Front Bioeng Biotechnol. 2020 Sep 15;8:568186. doi: 10.3389/fbioe.2020.568186 (PMC7523645; doi:10.3389/fbioe.2020.568186)
Supplement: Supplementary file 1 [file Image_1.pdf]

Supplementary Material

1 Supplementary Figures

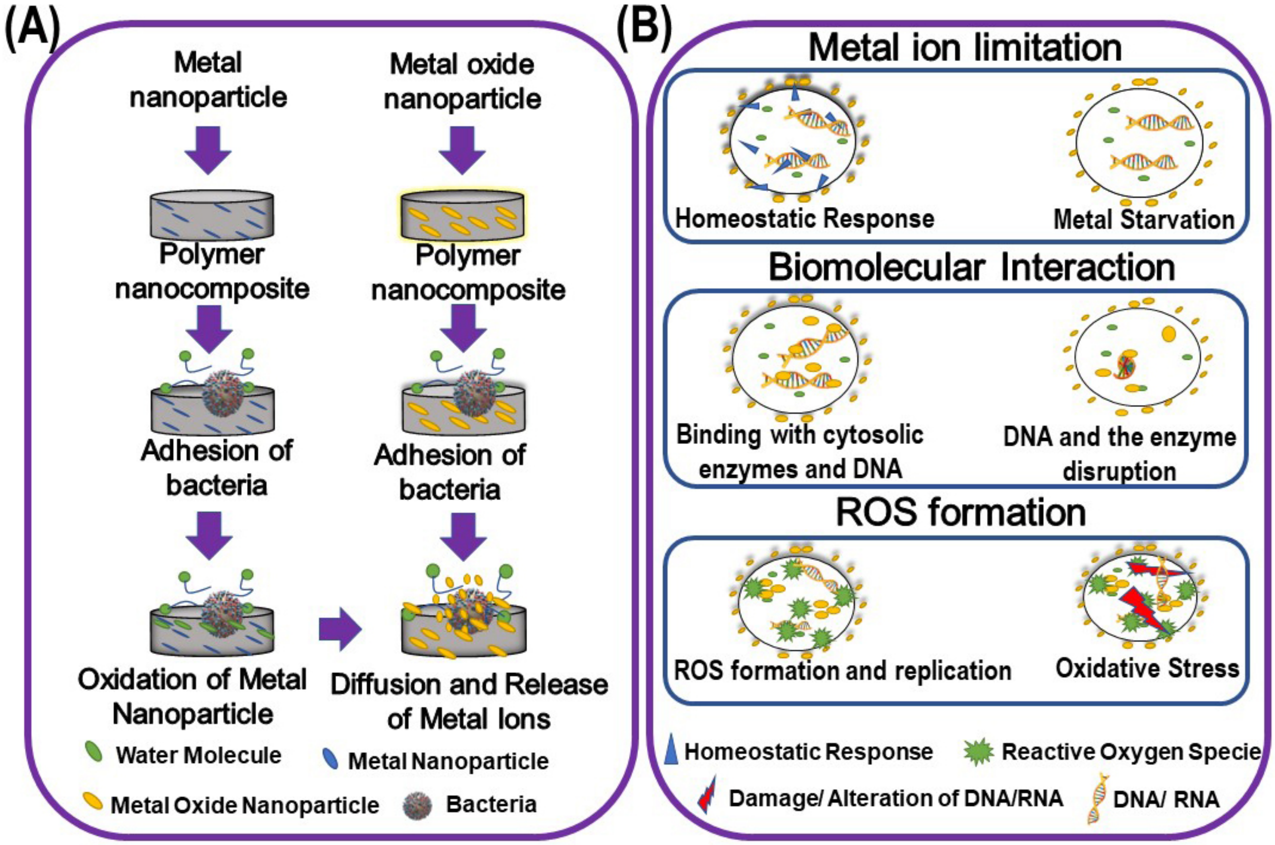

Figure S1. Antibacterial mechanism of M/MO incorporated thermoplastic nanocomposites. (A) Release mechanism of ions from the M/MO nanoparticles embedded into the thermoplastics as a response to bacterial adhesion on the nanocomposite surface. When the bacteria attach to the composite, water molecules diffuse from the bacterial medium into M/MO nanoparticles. Metal nanoparticles ionize or oxidize in presence of water, dissolved oxygen and reach to the bacteria surface. Direct release can be achieved for metal oxide or ionized metal nanoparticle. (B) Three different mechanism behind antibacterial properties of metal ions released from the M/MO nanoparticles, which

are metal ion limitation, biomolecular interaction, reactive oxygen species (ROS) formation respectively.

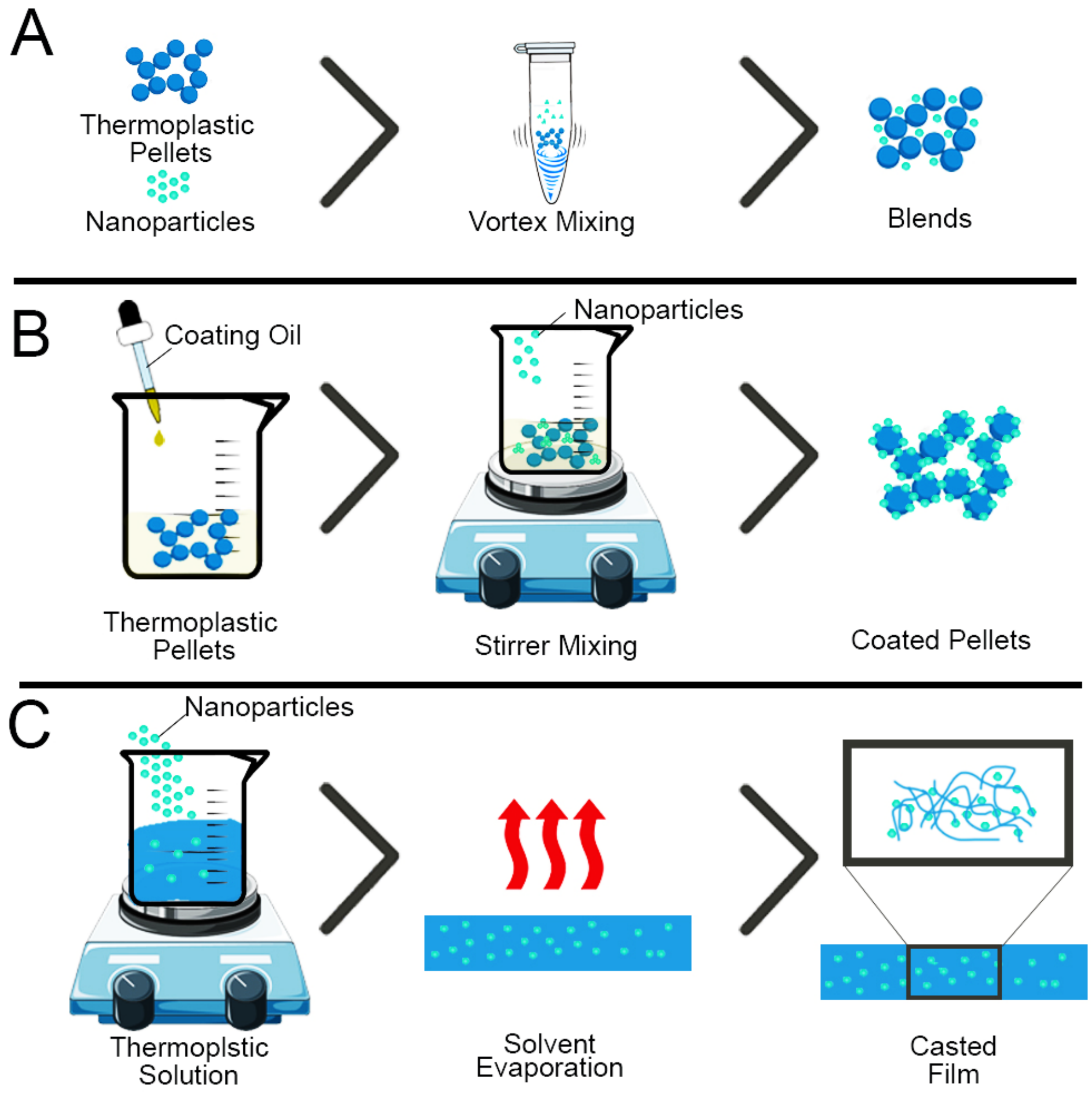

Figure S2. Different approaches to mix the nanoparticles with thermoplastics prior to filament processing. (A) Blending, (B) Surface coating, and (C) Solvent casting.
